# Disruption of Lipid Rafts Interferes with the Interaction of *Toxoplasma gondii* with Macrophages and Epithelial Cells

**DOI:** 10.1155/2014/687835

**Published:** 2014-03-09

**Authors:** Karla Dias Cruz, Thayana Araújo Cruz, Gabriela Veras de Moraes, Tatiana Christina Paredes-Santos, Marcia Attias, Wanderley de Souza

**Affiliations:** ^1^Laboratório de Ultraestrutura Celular Hertha Meyer, Instituto de Biofísica Carlos Chagas Filho, Universidade Federal do Rio de Janeiro, 21941-902 Rio de Janeiro, RJ, Brazil; ^2^Instituto Nacional de Metrologia e Qualidade Industrial-Inmetro, 25250-020 Duque de Caxias, RJ, Brazil

## Abstract

The intracellular parasite *Toxoplasma gondii* can penetrate any warm-blooded animal cell. Conserved molecular assemblies of host cell plasma membranes should be involved in the parasite-host cell recognition. Lipid rafts are well-conserved membrane microdomains that contain high concentrations of cholesterol, sphingolipids, glycosylphosphatidylinositol, GPI-anchored proteins, and dually acylated proteins such as members of the Src family of tyrosine kinases. Disturbing lipid rafts of mouse peritoneal macrophages and epithelial cells of the lineage LLC-MK2 with methyl-beta cyclodextrin (M**β**CD) and filipin, which interfere with cholesterol or lidocaine, significantly inhibited internalization of *T. gondii *in both cell types, although adhesion remained unaffected in macrophages and decreased only in LLC-MK2 cells. Scanning and transmission electron microscopy confirmed these observations. Results are discussed in terms of the original role of macrophages as professional phagocytes versus the LLC-MK2 cell lineage originated from kidney epithelial cells.

## 1. Introduction

*Toxoplasma gondii*, one of the most widely distributed pathogenic protozoa, is highly competent at invading a variety of cell types from different animals (Reviews in [1, 2]). Studies conducted by several groups over the last twenty years have provided a substantial amount of information on the roles of the proteins that the parasite secretes once it attaches to the host cell surface. These proteins trigger a series of events that culminate in the penetration of the host cell by the parasite through a typical endocytic process that includes the formation of a vacuole known as the parasitophorous vacuole (PV) [3–6]. Several proteins present in the micronemes and rhoptries and two major secretory organelles found in the apical portion of the protozoan, have been shown to play important roles in the interaction process [7, 8]. However, relatively little is known about the role of the host cell surface components during the parasite-host interaction [9–12]. Because* T. gondii* is able to penetrate all of the host cells tested, it is highly likely that molecular assemblies that are conserved in different cell types are involved in the parasite-host cell interaction process. One of these well-conserved machineries is the so-called “lipid rafts,” which are membrane microdomains that contain high concentrations of cholesterol, sphingolipids, glycosylphosphatidylinositol, GPI-anchored proteins, dually acylated proteins such as members of the Src family of tyrosine kinases, and so forth (reviews in [13–15]). Previous studies have shown that disturbing host cell lipid rafts by using drugs that interfere with cholesterol, such as methyl-beta cyclodextrin (M*β*CD) and filipin, or lidocaine, which does not interfere with cholesterol, significantly inhibited the infection of the cells by the protozoa* Leishmania donovani* [16],* Leishmania chagasi* [17],* Trypanosoma cruzi* [18, 19], and* Plasmodium falciparum* [20]. In the case of* T. gondii*, Coppens and Joiner [21] showed that depletion of the host cell membrane cholesterol using lovastatin or M*β*CD reduced parasite internalization and increased the number of parasites attached to the host cell surface.

To determine the role of membrane lipid microdomains in the interactions between protozoa and host cells, we used professional phagocytic cells (macrophages) and an epithelial cell line (LLC-MK2) in conjunction with several compounds that interfere with lipid microdomains to analyze the effect of these compounds on parasite-host interactions. The cells were visualized by electron microscopy, and the obtained results are reported here.

## 2. Materials and Methods

### 2.1. Chemicals

Methyl-*β*-cyclodextrin (M*β*CD), filipin III, *β* subunit of the cholera toxin (CTB), and lidocaine were obtained from Sigma-Aldrich Chemical Laboratory, USA. Stock solutions of M*β*CD, CTB, and lidocaine were diluted in water and filipin was diluted in dimethylsulfoxide (DMSO).

### 2.2. Parasites

The RH strain of* Toxoplasma gondii* was maintained by intraperitoneal passage into mice as described elsewhere [22].

### 2.3. Host Cells

The epithelial cell line LLC-MK_2_ (ATTC) and mouse peritoneal macrophages were used in this study. The cells were cultured in RPMI 1640 (Gibco) medium supplemented with 10% fetal bovine serum and maintained at 37°C in a 5% CO_2_ atmosphere. The macrophages were prepared and maintained as described previously [19].

### 2.4. Host Cell-Parasite Interaction

The interaction experiments were carried out with cells plated on 13 mm glass slides. Either the cells or the parasites were incubated in the presence of the various compounds tested, as indicated in the Results section. The parasite-to-host cell ratio was adjusted to 50 : 1. After the cells were allowed to interact, the host cells were washed to remove the unattached parasites and were then fixed in freshly prepared 4% formaldehyde in 0.1 M phosphate buffer, pH 7.2. After fixation, the cells were washed and stained with Giemsa, and the coverslips were dehydrated in acetone-xylol and mounted on glass slides with Entellan mounting media for subsequent observation with a light microscope (Carl Zeiss Microscopy GmbH, Jena, Germany). The adhesion and internalization indices were determined as described previously [23]. At least three independent experiments in duplicate were performed, and at least 600 cells were analyzed on each coverslip. The data obtained in the control experiments were normalized to 100. Graphic and statistical analyses, including Student's *t*-test and one-way ANOVA, were conducted with Prisma Graph Pad software (GraphPad Software).

### 2.5. Cell Viability Assay

After incubation with one of the drugs, the cells were rinsed in PBS and incubated in the presence of 0.2% Trypan blue for 5 minutes. The percentage of labeled cells (only dead cells are labeled) was determined by microscopic examination of at least 300 cells in at least three independent experiments.

### 2.6. Fluorescence Microscopy

To examine the localization of the GM1 ganglioside, the cells were washed in RPMI 1640 medium (GIBCO, Life Technologies Corporation) and incubated in the presence of 50 *μ*g/mL of the *β* subunit of cholera toxin (Sigma-Aldrich, USA) for 45 minutes. Subsequently, the cells were washed with PBS, pH 8.0, and incubated in the presence of 5 *μ*g/mL DAPI (Sigma-Aldrich, USA) to label the cell nuclei. After incubation, the cells were washed, mounted on glass slides with 0.2 M N-propyl gallate in 90% glycerol, and observed using a Zeiss Axioplan fluorescence microscope (Carl Zeiss Microscopy GmbH, Jena, Germany).

### 2.7. Scanning Electron Microscopy

After interacting with the parasites, the host cells mounted on coverslips were fixed in a solution containing 2.5% glutaraldehyde in 0.1 M cacodylate buffer, pH 7.2, for 1 hour, washed in buffer, and postfixed for 30 minutes in the dark in a solution containing 1% osmium tetroxide and 0.8% potassium ferrocyanide in the same buffer. The cells were washed again in buffer, dehydrated in ethanol, and submitted to critical point drying in CO_2_ using CPD 30 Baltec equipment. The samples were then coated with a 0.2 nm thick layer of gold and examined in a Jeol JSM 6340 field emission scanning electron microscope operating at 3–5 kV.

### 2.8. Transmission Electron Microscopy

After interacting with the parasites, the host cells were fixed for 1 hour at 4°C in a solution containing 2.5% glutaraldehyde and 1.4% freshly prepared formaldehyde in 0.1 M cacodylate buffer, pH 7.2. Then, the cells were washed in buffer and postfixed for 40 minutes in the dark in a solution containing 1% osmium tetroxide and 0.8% potassium ferrocyanide in 0.1 M cacodylate buffer, pH 7.2. The cells were then washed in buffer, dehydrated in acetone, and embedded in an Epoxy resin. Thin sections were obtained using an ultramicrotome. The sections were stained with uranyl acetate and lead citrate and examined using a transmission electron microscope (Zeiss 900 or Jeol 1200) operating at 80 kV.

## 3. Results

### 3.1. M*β*CD Treatment of the Host Cell Interferes with the Interaction Process

**Figure 1:**
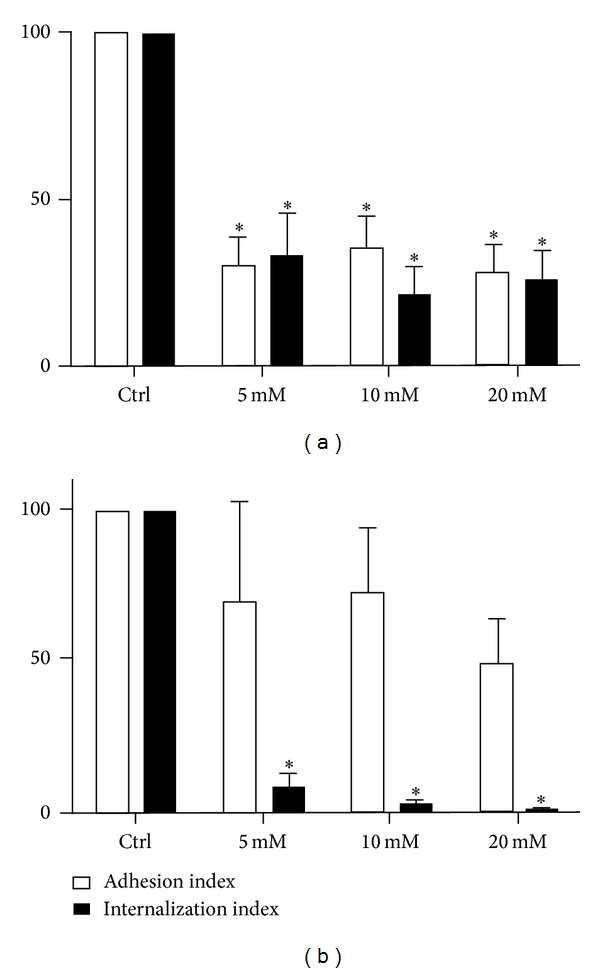
*T. gondii* adhesion and internalization percentages with LLC-MK2 cells (a) and murine macrophages (b) that were treated with M*β*CD (5, 10, and 20 mM) for 30 min before the addition of* T. gondii *tachyzoites. The host cells were pretreated and washed before interacting for 10 minutes with the parasites (50 : 1). The data shown are the means ± SE of duplicated points from three independent experiments. The control values were set at 100. In LLC-MK2 cells, both adhesion and internalization were inhibited. However, only internalization was affected in macrophages.

**Figure 2:**
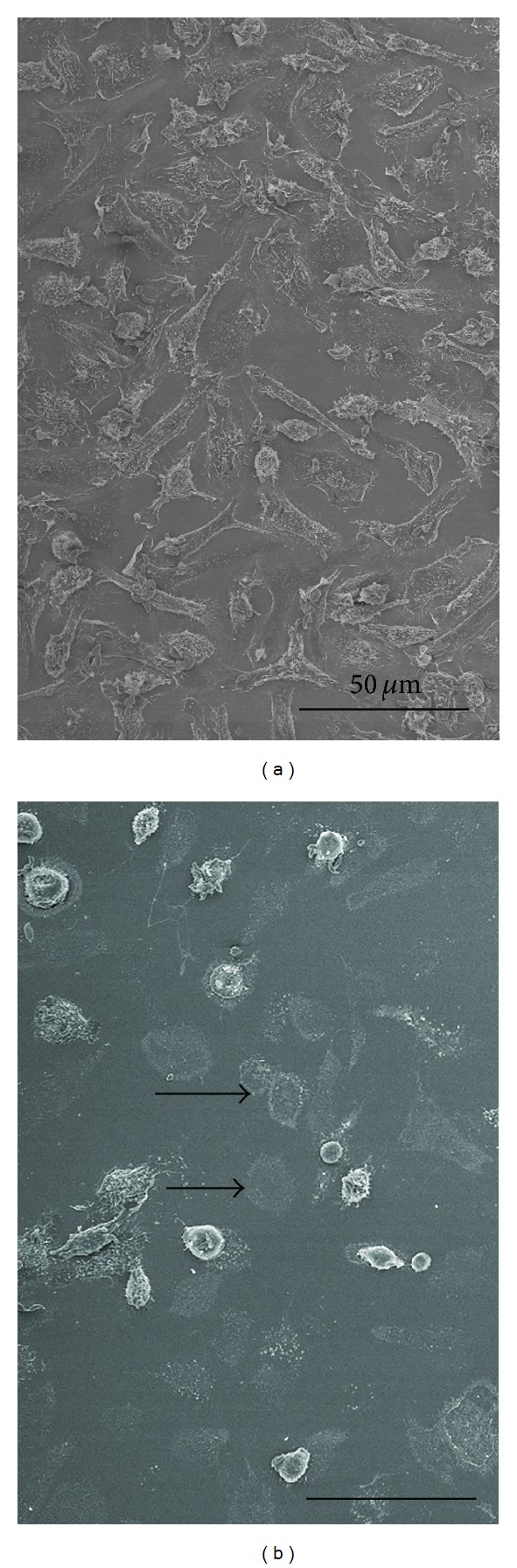
Scanning electron microscopy images of control macrophages (a) evenly spread over the glass surface. (b) Treatment with 20 mM M*β*CD caused the macrophages to detach from the glass, leaving a print in the coverslip (arrows). Macrophages that resisted the treatment were more spherical, suggesting a retraction of filopodia and adhesion points. Bar: 50 *μ*m.

**Figure 3:**
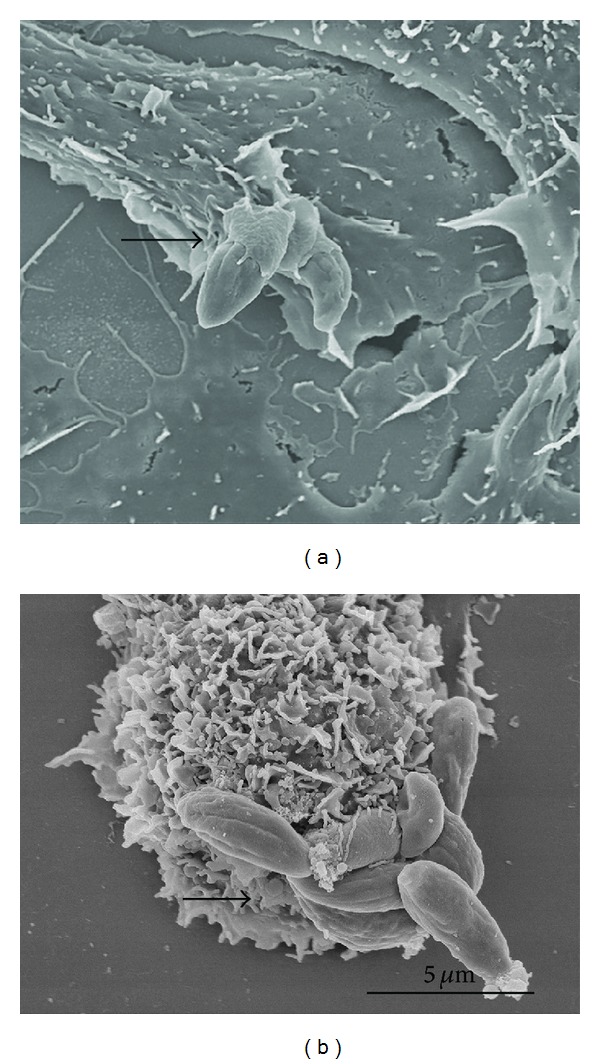
Scanning electron microscopy images of untreated macrophages (a) and two parasites partially covered by membrane projections, indicating that they are undergoing internalization (arrow). (b) Eight parasites (arrow) can be seen adhered to this macrophage that was previously treated with 20 mM M*β*CD.

**Figure 4:**
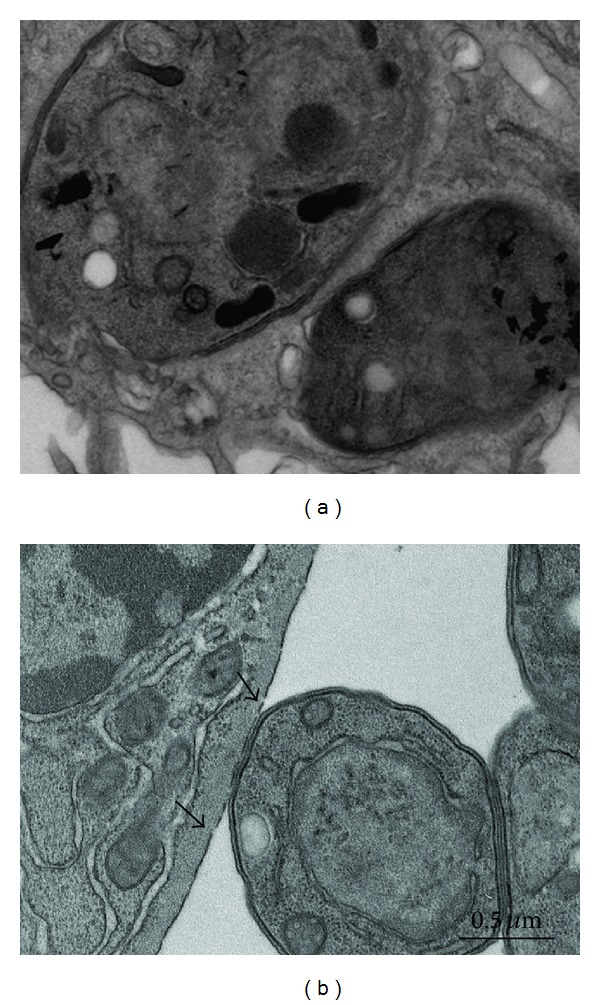
Transmission electron microscopy images of a control (untreated) macrophage (a) containing two parasites. (b) A single parasite is seen adhered to the surface of a cell that had been pretreated with 20 mM M*β*CD. The plasma membrane shows a dotted pattern (arrowheads).

We used M*β*CD, a cyclodextrin that is a glucose oligomer that interacts with membranes and sequesters lipophilic molecules within its hydrophobic nucleus [13]. Treatment of LLC-MK2 cells with M*β*CD followed by incubation with* T. gondii* significantly decreased (*P* < 0.0001) both the adhesion and the internalization indices (Figure 1(a)). Inhibition was evident even at a concentration of 5 mM M*β*CD, reaching values as high as 70%. Inhibition did not increase when higher concentrations of M*β*CD were used. In macrophages, only a slight inhibition of the adhesion index was observed. However, internalization was drastically reduced by up to 95% by treatment with 5 mM M*β*CD (Figure 1(b)). Notably, treatment of both cells types with M*β*CD at the concentrations used did not significantly interfere with cell viability, as determined using Trypan blue (data not shown). Scanning electron microscopy of M*β*CD-treated macrophages revealed that the cells became more retracted and rounded. Some cells detached from the glass surface such that the number of cells per square micrometer was significantly decreased, as shown in Figure 2. However, the detached cells were still viable (data not shown). The parasites attached to untreated macrophages triggered endocytic processes, and membrane projections were observed surrounding the parasites during the process of internalization (Figure 3(a)). In contrast, no surface membrane projections were observed surrounding the parasites attached to the surface of M*β*CD-treated macrophages (Figure 3(b)). Transmission electron microscopy observations confirmed that parasites were internalized by untreated host cells (Figure 4(a)) but they remained attached to the surface of M*β*CD-treated cells (Figure 4(b)).

**Figure 5:**
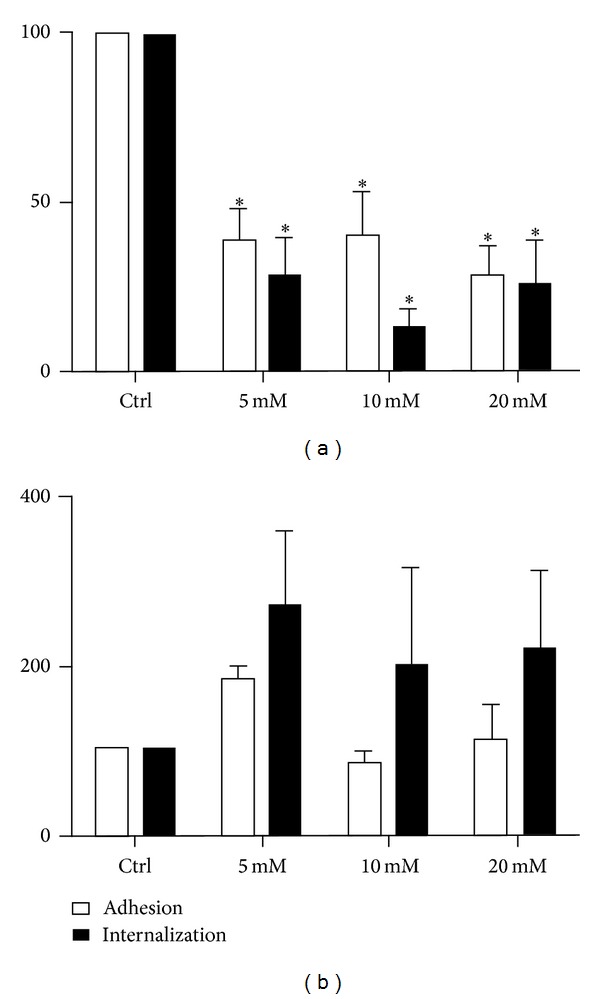
Reversibility of the effect of M*β*CD. Estimation of adhesion and internalization percentage of* T. gondii* with LLC-MK2 cells (a) and murine macrophages (b) treated with M*β*CD (5, 10, and 20 mM) for 30 min and then incubated for 2 hours with 20% FCS in RPMI medium before the* T. gondii *tachyzoites were added for 10 minutes (50 : 1). The data shown are the means ± SE of duplicated points from three independent experiments. *P < 0.05. The results were normalized. Reversibility was observed only in macrophages.

We also analyzed the reversibility of the M*β*CD treatment. For this experiment, the cells were initially treated with M*β*CD for 30 minutes. Subsequently, some cultures were washed and incubated in fresh medium for 2 hours before interaction with the parasites. No reversibility was observed with the LLC-MK2 cells (Figure 5(a)). However, a significant reversible effect on the internalization of the parasites by macrophages was achieved (Figure 5(b)), but this effect was less evident for the adhesion index. Scanning electron microscopy showed that the recovered macrophages possessed surface projections that covered the attached parasites, similar to what was observed in the control (not shown).

### 3.2. Filipin Treatment of Host Cells Interferes with Their Interaction with* T. gondii*

**Figure 6:**
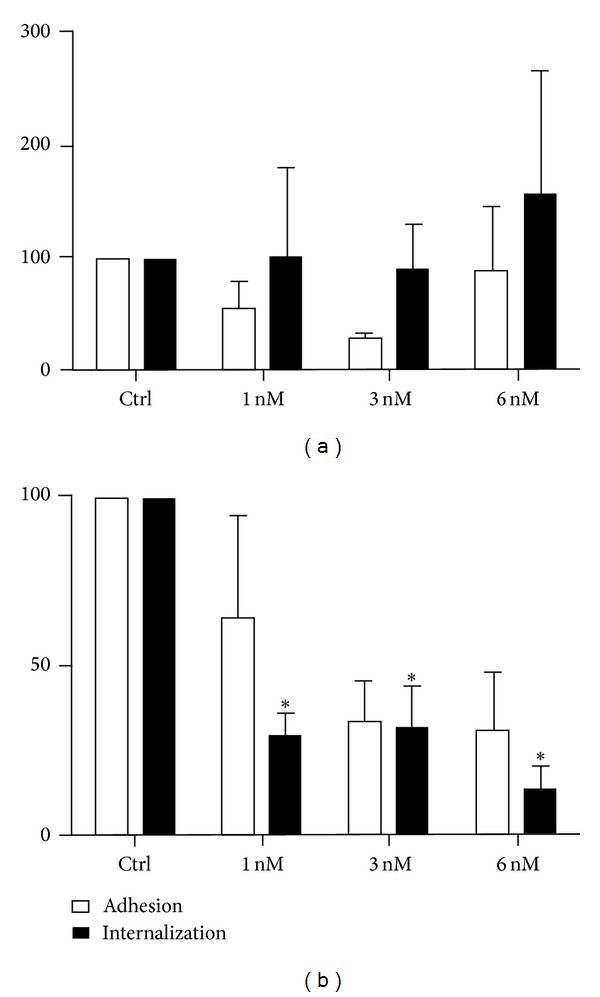
Adhesion and internalization percentages with (a) LLC-MK2 cells and (b) murine macrophages after filipin treatment (1, 3, and 6 nM) for 30 min before the addition of the parasites (50 : 1) for 10 minutes. At 6 nM filipin, the adhesion of the parasites to the LLC-MK2 cells was slightly decreased, and internalization was significantly inhibited. In macrophages, internalization was inhibited by 85%. The data shown are the means ± SE of duplicated points from three independent experiments. *P < 0.05. The results were normalized.

Filipin is a polyenic antibiotic that binds to cholesterol and thus interferes with the fluidity of cell membranes. Therefore, we decided to analyze its effect on the* T. gondii*-host cell interaction. Incubation of the LLLC-MK2 cells in the presence of 1 or 3 nM but not 6 nM filipin slightly decreased the adhesion of the parasites to the cells (Figure 6(a)) but did not interfere with internalization. In macrophages (Figure 6(b)), filipin inhibited both adhesion and internalization. At a filipin concentration of 6 nM, internalization was inhibited by 85%. In the tested conditions, the treated cells remained viable (data not shown). Scanning and transmission electron microscopy confirmed these observations (data not shown).

### 3.3. Treatment of the Host Cells with the B Subunit of the Cholera Toxin Inhibits Parasite-Host Cell Interaction

**Figure 7:**
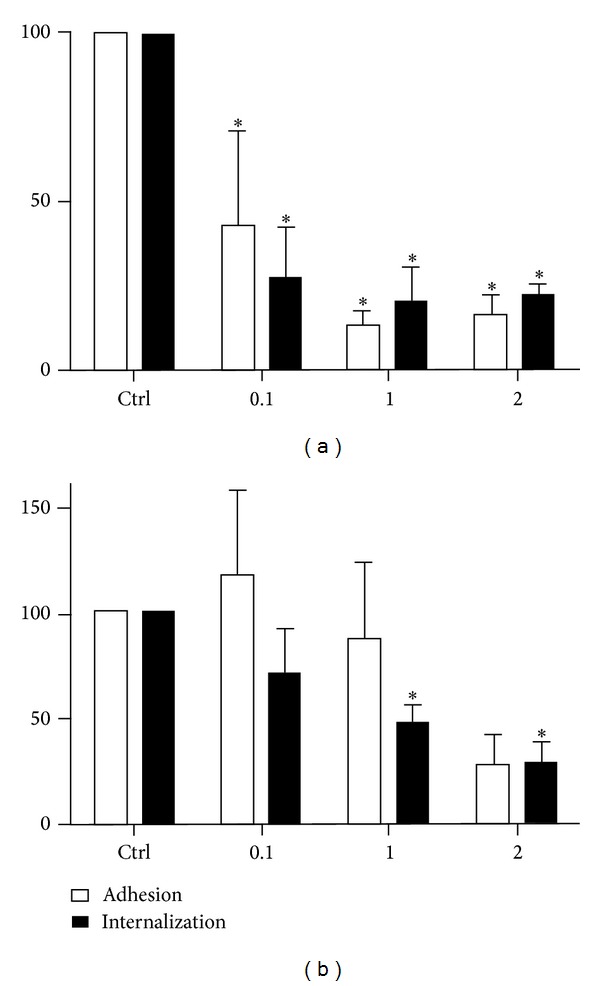
Adhesion and internalization indices of* T. gondii* in LLC-MK2 cells (a) and murine macrophages (b) after treatment with cholera toxin-B (CTB) (0.1, 1, and 2 *μ*g/mL) for 20 minutes at 4°C before the addition of parasites (50 : 1) at 37°C for 10 minutes. The cells were then fixed and stained. In LLC-MK2 cells, adhesion and internalization were reduced at all concentrations tested, and in macrophages, a significant reduction in internalization was observed at the higher concentrations. Parasite loads were quantified microscopically, and the data shown are the means ± SE of duplicated points from three independent experiments. *P < 0.05. The results were normalized.

We also analyzed the influence of the GM1 ganglioside on the interaction process. For this experiment, the cells were treated for 30 min at 4°C with the *β* subunit of cholera toxin (CTB) and then allowed to interact with parasites at 37°C. Treatment with CTB greatly inhibited the adhesion to and invasion of LLC-MK2 cells and reached inhibition values of 80% (Figure 7(a)). The macrophages treated with CTB also showed altered adhesion and internalization indices (Figure 7(b)). Treatment of the cells with CTB did not affect their viability (data not shown).

### 3.4. Treatment with Lidocaine Interferes with* T. gondii*-Host Cell Interaction

**Figure 8:**
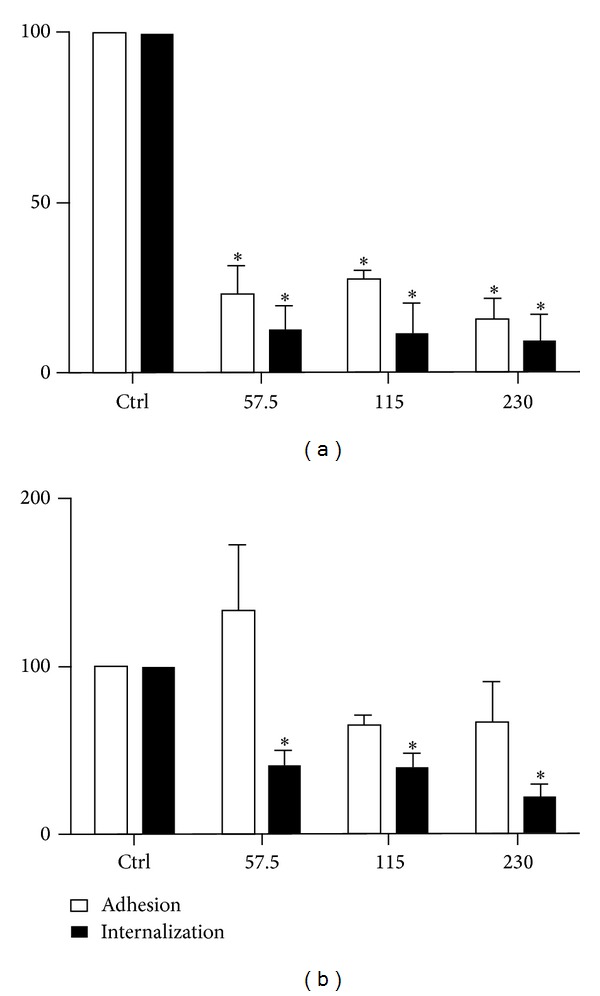
Estimation of the adhesion and internalization indices of* T. gondii* in LLC-MK2 cells (a) and murine macrophages (b) pretreated with lidocaine (57.5 *μ*M, 115 *μ*M, and 230 *μ*M) for 20 minutes before the addition of parasites at 37°C for 10 minutes. In LLC-MK2 cells, both adhesion and internalization were reduced at all concentrations. In macrophages, a significant reduction was observed only for internalization. The parasite loads were quantified microscopically. The data shown are the means ± SE of duplicated points from three independent experiments. *P < 0.05. The results were normalized.

We used lidocaine in our experiments because it is able to penetrate the membrane lipid bilayer and can disrupt the lipid rafts [24]. We selected concentrations of lidocaine ranging from 57.5 to 230 *μ*M and incubated the cells with the lidocaine for 20 minutes at 37°C. After incubation, the cells were washed in media and allowed to interact with the parasites. We observed that the lidocaine treatment markedly inhibited the adhesion and internalization of the parasites incubated with LLC-MK2 cells by up to 90% (Figure 8(a)). The same treatment interfered to a lesser extent with parasite adhesion to the macrophages, but it significantly inhibited internalization (Figure 8(b)). Cell viability tests indicated that the cells remained viable after incubation with lidocaine (data not shown).

### 3.5. Effect of Pretreating* T. gondii *with M*β*CD, Filipin, CTB, or Lidocaine on the Interaction with Host Cells

Experiments were performed to determine if the same compounds tested in the host cells also affected membrane domains on the surface of* T. gondii*.  Table 1 summarizes the observations made from these experiments. It is important to note that the effects observed from treating the parasites were not as clear as those obtained following treatment of the host cells. In most of the experiments, we observed a reduction in the internalization index with a much slighter effect on the adhesion index.

## 4. Discussion

The concept that the cell membrane is more mosaic than fluid with nonrandom distribution of lipids was a major step in understanding the behavior of cells, particularly cell interaction with pathogens (review in [25]). In basal conditions, lipid rafts are small regions of the membranes. However, they can form larger clusters in response to certain stimuli [26, 27]. Data obtained by several groups in the last decade have established that the interaction of intracellular pathogenic protozoa with host cells involves two well-defined steps: adhesion and internalization (reviews in [24, 28–30]). Adhesion can occur even at low temperatures or when the host cell cytoskeleton is blocked by the use of drugs, and the participation of molecules that are exposed on the surface of both interacting cells is very important to this process [31]. The internalization process involves cell signaling events followed by endocytosis [29], and the composition of the plasma membrane and the mobility of membrane-associated molecules, which depends on the fluidity of the lipid bilayer and association with cytoskeletal components, play an important role.

It is well established that lipid rafts in the membrane of the host cell are involved in the internalization of pathogenic protozoa such as* Leishmania* [16],* Trypanosoma cruzi* [18, 19],* Plasmodium falciparum* [20], and* Toxoplasma gondii* [21]. The moving junction, a specialized contact area established by* Plasmodium* during invasion of an erythrocyte [32] and between* Toxoplasma* and any host cell it invades [33], selects host cell rafts and proteins that will be part of the parasitophorous vacuole membrane [34]. However, caveolin I, a typical raft-associated protein, is excluded from the parasitophorous vacuole of* T*.* gondii* [33], as well as flotillin-2 [35]. In this study with* T. gondii*, we further analyzed the role of membrane lipid domains, including lipid rafts, in the interaction process using LLC-MK2 cells and murine macrophages and treatment with several inhibitors. Our results were visualized by electron microscopy to obtain a more detailed view of the parasite-host cell interface.

We used M*β*CD, filipin, the *β* subunit of cholera toxin, and lidocaine to interfere with the membrane of the host cell. These various treatments affected both the shape of the cells and their ability to adhere to the glass coverslips. However, cell viability was not significantly altered, as assessed using the Trypan blue test.

M*β*CD is a glucose oligomer that sequesters lipophilic molecules in hydrophobic nuclear membranes [13], and it has been widely used to deplete cholesterol from membranes and prevent endocytic processes. Treatment of host cells with M*β*CD has been shown to decrease the internalization of* Leishmania* [16],* T. cruzi* [18, 19], and* T. gondii *[21]. In* Plasmodium*, depletion of cholesterol from the membranes of infected erythrocytes caused precocious liberation of parasites, which were noninfective [34]. Red blood cells depleted from cholesterol also prevent invasion by* Plasmodium falciparum* [36]. Our observations confirm the results previously described for these protozoa, but we also were able to show that the effects of the treatment varied according to the host cell used. Indeed, M*β*CD treatment only slightly interfered with the adhesion of* T. gondii* to macrophages; however, it inhibited adhesion to LLC-MK2 cells by 75%. A similar effect was reported for fibroblasts and CHO cells [21]. Notably, in the case of* T. gondii*, M*β*CD interfered acutely with parasite internalization at concentrations as low as 5 mM, while similar effects on* T. cruzi *were obtained with 20 mM [19]. In malaria parasites, also apicomplexans, erythrocyte lipid rafts are recruited to the site of invasion and can be remodeled by* Plasmodium* to establish blood stage infection [37].

We used scanning and transmission electron microscopy to analyze the interaction process in untreated control cells and drug-treated cells. Our observations show clearly that host cells treated with M*β*CD or with the other tested drugs had parasites which adhered to the cell surface, but, unlike the untreated cells, no host cell surface projections surrounded the parasites. Therefore, interfering with the plasma membrane lipids of the host cell blocks the formation of surface projections.

Filipin is another compound that has been used to interfere with membrane cholesterol. Unlike M*β*CD, this polyenic antibiotic does not extract cholesterol but instead binds to it and forms filipin-sterol complexes that drastically decrease the fluidity of the plasma membrane [30]. Filipin inhibited the internalization of* T. gondii* by macrophages by up to 85%, but it did not have a significant effect on the interaction of the parasite with LLC-MK2 cells. We previously reported that filipin had a discrete effect on both the adhesion and internalization of* T. cruzi *[19].

We also tested the effect of lidocaine on host-parasite interactions. Lidocaine is a local anesthetic that disrupts lipid rafts without altering membrane cholesterol [38] and has been shown to inhibit the infection of erythrocytes by* Plasmodium falciparum* by 90% [39]. Treatment of host cell with lidocaine markedly inhibited both the adhesion and internalization of* T. gondii* by LLC-MK2 cells but only decreased internalization in macrophages.

Previous studies have shown that treating host cells with the *β* subunit of cholera toxin, which is produced by the bacterium* Vibrio cholera* and binds to the GM1 ganglioside, a well-known marker of lipid rafts [17], markedly blocked the internalization of* T. cruzi* by macrophages [19]. A similar effect was observed both for the adhesion and internalization steps in the interaction of* T. gondii *with LLC-MK2 cells and for its internalization into macrophages. Surprisingly, it had only a small effect on the attachment of* T. gondii* to macrophage surfaces.

Taken together, the available data clearly show that the organization of the lipid rafts in the host cell plasma membrane is essential for the initiation of the endocytosis that leads to the internalization of* T. gondii*, even when cholesterol is present in the plasma membrane. The differences observed between adhesion and internalization indices between macrophages and LLC-MK2 cells could be explained by the very nature of these cell types. Macrophages are professional phagocytes and internalization was not blocked as efficiently as for LLC-MK2 cells. That could be due to the fact that the observed internalization was the result of phagocytosis, rather than active invasion by the parasites. Adhesion indices on the other hand were reduced in both cell types, indicating that raft disruption impairs this key step in parasite active invasion of host cells.

We also carried out experiments to determine whether treating the parasite with the same compounds discussed above also interfered with the interaction process. We observed decreased internalization in most of the experiments, but they had a high standard deviation that was most likely due to the heterogeneity of the parasites used, which were obtained from the peritoneum of experimentally infected mice. Furthermore, the very intense and continuous secretion of micronemes constantly renews the outer membrane of the parasite. These observations suggest that the organization of the lipid bilayer of the parasite also plays a role in the process of adhesion to and internalization by host cells. Previous studies have suggested that an interchange of surface components of the two cells involved in the interaction process occurs [40].

## Figures and Tables

**Table 1:** Effect of drugs that interfere with lipid rafts on *Toxoplasma gondii* adhesion and internalization of host cells.

Cell type	Macrophages		LLC-MK2	
Treatment	Adhesion	Internalization	Adhesion	Internalization

M*β*CD	Nonaffected	Decreased	Decreased	Decreased
Filipin	Nonaffected	Decreased	Nonaffected	Nonaffected
CTB	Nonaffected	Decreased	Decreased	Decreased
Lidocaine	Nonaffected	Decreased	Decreased	Decreased
